# The Formulation of Cheese Analogue from Sweet Corn Extract

**DOI:** 10.1155/2019/8624835

**Published:** 2019-05-02

**Authors:** Nur Aini, Vincentius Prihananto, Budi Sustriawan, Didik Romadhon, Riza N. Ramadhan

**Affiliations:** Department of Food Science and Technology, Jenderal Soedirman University, Purwokerto, 53123, Indonesia

## Abstract

Analogue cheese made from sweet corn extract was expected to fulfill the people's need for cheese and as alternative cheese made from cow's milk. The use of maltodextrin as a filler and citric acid as an acidulant was expected to improve the characteristics of corn cheese. The aims of this article were to (1) determine the optimum concentration of maltodextrin, papain, and citric acid in order to produce corn milk-based cheese analogue with the best characteristics; (2) determine the characteristics of cheese analogue produced using the optimum concentration. The research design used in this study was Response Surface Methodology (RSM) based on Central Composite Design (CCD) with three factors: citric acid concentration (0.12%, 0.16%, and 0.20%), commercial papain (0.026%, 0.030%, and 0.034%), and maltodextrin (10%, 15%, and 20%). The optimum formula to produce cheese analogue with the highest protein content and yield was with the addition of 0.20% citric acid, 0.029% papain, and 20% maltodextrin. The cheese analogue produced from the optimum formula had moisture content of 61.590%, yield of 17.512%, total dissolved solids of 19.00°Brix, dissolved protein of 19.837%, acidity (pH) of 5.4, and fat of 6.976%. The sensory characteristics of cheese analogue spread from sweet corn extract are similar to those of cheese from cow's milk; i.e., it had a yellowish-white color, distinctive aroma of cheese, no sour taste, and soft texture and was easy to spread. Therefore, it was possible to explore the sweet corn as ingredient of spread cheese that has low fat content.

## 1. Introduction

Milk and dairy products have high nutritional value that contributes to fulfilling nutrient and protect against chronic diseases [1]. However, [2] states that milk fat contains over 70% saturated lipids which are associated with an increased risk of heart and artery disease. To get the right benefits of the fat consumed, it is considered equally important to the balance of saturated to unsaturated fatty acids. Substitution of milk fat with vegetable fat is an alternative to obtain dairy products with a balanced saturated/unsaturated fat. One of the dairy products that can be substituted with the other ingredients is cheese, whose product is called cheese analogue.

Cheese analogue consists of milk protein and nondairy and substitute oil or milk fat as a substitution for milk solid. The advantage of cheese analogue was the production cost which was relatively lower compared to cheese made from cow's milk [3]. The studies on cheese analogue that had been conducted were on, for example, cheese analogue made from soybeans [4], pectin gel [5], inulin [6], rice flour [7], apricot pulp [8], and replacement of fat types [9]. It is proven that vegetables, fruit, or cereals can be used as substitutes in making cheese analogue [10]. Sweet corn can be processed into extract that has milk-like taste, which can then be used to substitute for dairy products, for example, into corn yogurt [11]. Thus, sweet corn also has the potential to substitute in making cheese analogue. So far, there has been no research conducted on cheese analogue made from sweet corn extract. In this study, we make analogue spread cheese because it is easy and fast to make.

Production of cheese requires a coagulation process that usually uses the rennet enzyme [12]. However, the production of rennet enzyme was quite low due to the mammals' regeneration system and it took quite some time [13]. Thus, the price of rennet is expensive. Therefore, it was necessary to find alternative source to other types of protease enzyme with cheaper prices. Protease enzyme can be used as coagulant in cheese making, for example, from latex [14], bromelin from pineapple [15], and lime and star fruit [16]. In this study, papain was used as coagulant because it was easy to obtain and cheap.

In the cheese production, it was necessary to add acidic ingredients that would assist the coagulation process by lowering the pH and determine the cheese texture. One of the acidic ingredients that could be used was citric acid [17]. The use or addition of citric acid could accelerate the coagulation of casein which would affect the protein content of the cheese produced. According to [18], the use of 1% citric acid as coagulation agent will produce mozzarella cheese from buffalo milk with a yield of 15.12%.

The making of cheese without ripening generally would produce a small amount of yield [19]. This was because the coagulation process of curd occurred in a short time and it made many solids dissolve in the whey. The attempt to increase the cheese yield could be done by adding filler during the cheese making process [20]. Maltodextrin is used as filler in this study because it is cheap and the yield is high.

The aims of this study were to (1) determine the optimum concentration of citric acid, papain, and maltodextrin to produce corn milk-based cheese analogue which had the highest yield; (2) determine the chemical and sensory characteristics of cheese analogue produced from the optimum formulation.

## 2. Materials and Methods

The main ingredients used in this study were sweet corn from Pasar Wage Purwokerto, Indonesia, cow's milk (from the Exfarm of Jenderal Soedirman University, Purwokerto),* Lactobacillus casei* (10^6^ cfu/g) purchased from Indonesian Institute of Sciences, papain, maltodextrin, and citric acid. Cow's milk (which has specific characteristics: pH 6.57, total protein 3.31%, density 1.028 g/cm^3^, titratable acidity 13.5%, and fat content 3.72%. The main tools used were blenders, presses, spectrophotometers (Shimano UV-VIS 1800), refractometer (Atago), pH meter, analytical balance (AND), and oven (Memmert).

### 2.1. Formulation and Characterization of the Initial Products

The experiment was conducted in three steps. The first step was the determination of the initial formula and characterizing the products' properties. The second was the determination of the optimum formula and its verification. The last step was the determination of the best product.

In first step, the initial formula was determined using the* Response Surface Methodology* (RSM) method based on* Central Composite Design* (CCD) with 3 factors, namely, citric acid concentration (0.12%, 0.16%, and 0.20%), papain concentration (0.026%, 0.030%, and 0.034%), and maltodextrin (10%, 15%, and 20%). Based on the* Design Expert* results, there were 20 formulas of cheese analogue that were tried. Each formula was tried and analyzed for its properties, such as moisture content [21], yield [19], dissolved protein [21], and acidity (pH) and fat content [21].

Moisture content was analyzed using the AOAC method in the following way: 2-gram sample is weighed and put in a bowl that has been dried. Then the sample and cup were dried in a 105°C temperature oven until a constant weight was obtained. Moisture content is the difference between the weight of the starting material and the weight of the final material after drying [21].

The yield is the ratio between the analogue cheese ingredients after processing with the material and before processing multiplied by 100% [19]. Yield calculation is based on the formula

(1) $$\begin{array}{c} Yield=\frac{cheese\;\;weight}{the\;\;weight\;\;of\;\;the\;\;constituent}\times 100\% \end{array}$$

Dissolved protein was analyzed by the method of Lowry-Folin in the following way: 0.1-1 g of sample dissolved in 100 ml of distilled water. Then 1 ml of the sample was taken and then put in a test tube. The sample was added with 5.5 ml of copper sulfate and left for 10-15 minutes at room temperature. Then add 0.5 ml of Folin Ciocalteau to each of the test tubes and immediately beat them evenly quickly. Then leave them for 30 minutes until the blue color is formed. The intensity of blue is measured by its absorbance using a spectrophotometer at a wavelength of 600nm. Calculation of dissolved protein levels was determined using the standard bovine serum albumin curve.

pH measurement was performed using a pH meter. However, before being used in cheese, the pH meter is calibrated beforehand with buffer solution pH 7.

Fat content measurements were carried out by using Soxhlet as follows: 2-5 grams of finely ground sample, wrapped in filter paper, inserted in Soxhlet extraction tube. Then a fat dish and extraction tube are installed on the distillation apparatus. Soxhlet which has been filled with solvents is then drained by cooling water and the appliance is turned on. Extraction is carried out for 4-5 hours. After that, the solvent is separated from the fat, while the fat-filled dish is dried in an oven at 100-105°C for 30 minutes. The residual weight in the fat dish is expressed as fat weight.

### 2.2. Process Optimization

The optimization process in second stage was done to obtain a formula with the most optimal response. The most optimal response was obtained if the desirability value approached 1 (one). The optimized components were research factors (concentration of citric acid, papain, and maltodextrin) and variables observed in stage I (moisture content, yield, soluble protein, pH, and fat). Each component's weight of interest was measured to achieve the desired goals. The relative importance of the component and the measured response was directly proportional to the weighting value given. It meant that the higher the relative importance of the component and the measured response, the greater the weighting value given. Data processing was done using Design Expert.

At the third step, the optimum formula (3 formulas) obtained from stage 2 was then analyzed for its sensory properties. Sensory analysis was done by examining the color, taste, aroma, texture, spread ability, and preference using a rank test. In testing the level of preference, trained panelists were invited to give their assessment. Out of the three optimum formulas, the best formula was then chosen.

### 2.3. Cheese Analogue Making

Cheese analogue was made using a modified method [22]. The stages in making cheese analogue were making corn extract, mixing the corn extract and cow's milk (1:1), pasteurization, cooling, addition of coagulant, and pressing and heating of the cheese. Making corn extract begins with steaming corn for 30 minutes, then blending it with water (corn: water = 1: 2) for 3 minutes. The blended corn is then filtered, so that corn extract is obtained. Corn extract is then mixed with cow's milk (1: 1), then pasteurized at 72°C for 30 minutes, and then cooled to 40°C. Furthermore 500 ml is taken and then added to 1 ml of* Lactobacillus casei*, 2 g of CaCl_2_, and 30 g of sugar, which is then stirred and allowed to stand at 40°C for 2 hours. The next stage is the addition of citric acid, papain, and maltodextrin, which is then allowed to stand for 30 minutes. To the milk that has undergone coagulation then 500 ml of hot water is added and then pressed. To the pressed milk 500 ml of hot water is then added and pressed (second pressing) for 30 minutes. The last step is heating the curd at 52°C for 15 seconds while adding 8% sugar and 2% salt.

## 3. Results and Discussion

### 3.1. Characterization of the Initial Product

**Figure 1:**
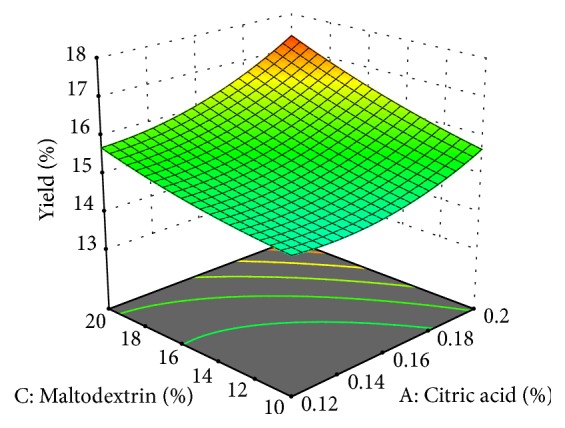
Yield of cheese analogue from sweet corn extract was affected by concentration of citric acid and maltodextrin.

The yield of cheese analogue was 14.262-17.072% (Figure 1). The yield is greater than the yield of cheese from cow's milk conducted by [18], which is of 6.4 - 7.0% with the same acidifying agent, namely, citric acid. This was because maltodextrin was added as filler in this study which also increased the yield.

An increase in the concentration of citric acid would decrease the cheese yield (Figure 1). According to [23], the higher addition of acid during cheese production by direct acidification would make the yield low. This was likely due to protein instability under acid condition during proteolysis. The more citric acid added would make the pH decrease. It would then result in higher proteolysis so that the amount of protein dissolved in liquid increase. Dissolved protein was inversely proportional to the cheese yield. Therefore, the higher dissolved protein, the fewer yields.

The addition of maltodextrin in the cheese analogue making increases the yield (Figure 1). Maltodextrin has a hygroscopic property which is able to absorb more water and increase the cheese yield. Maltodextrin also functions as filler to increase the volume, creating the cheese texture, as carrier and crystallization inhibitor [24].

The yield of cheese analogue was 14.262-17.072%. The addition of papain did not affect the yield. This result is slightly different from [25] which makes cheese using papain and the yield was higher (20.4-23.4%). According to [25], the higher amount of papain will increase the yield due to the enzymatic reactions. The difference between the results of this study and the previous is due to the differences in ingredients and the amount of papain. Papain used in this study was 0.026-0.034%, while in [25] the papain added is 1%.

These analogue cheeses have dissolved solid 17-20°Brix. The higher the addition of maltodextrin, the more the analogue cheese dissolved solids. With the presence of maltodextrin, the suspended particle will be trapped in the system and will not settle by the influence of the gravitational force.

The higher the concentration of citric acid, the more the dissolved solids. Citric acid can hydrolyze sucrose into glucose and fructose. The more the concentration of citric acid, the higher the glucose and fructose formed, so that dissolved solids of cheese analogues increase.

The higher concentration of papain added increases the total dissolved solids of cheese analogues. The higher the papain, the higher the proteolytic activity, so that the amino acid produced is also high. Amino acids are dissolved solids so that dissolved solids in analogue cheese increase.

**Figure 2:**
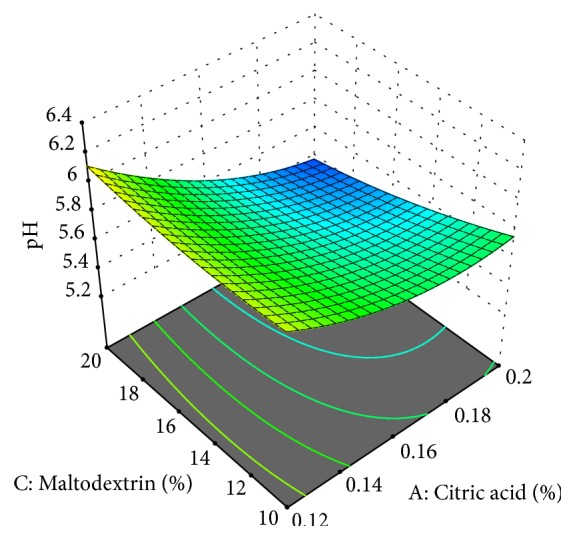
pH of cheese analogue from sweet corn extract was affected by concentration of citric acid and maltodextrin.

The pH of cheese analogue was 5.3-6.4 (Figure 2: pH of cheese analogue). The citric acid was inversely proportional to the pH of the cheese analogue. The more citric acid added to cheese analogue making, the lower pH of cheese analogue. This result is similar to [18] which states that increasing the amount of citric acid in cheese production will reduce the pH of the cheese analogue.

The increase of maltodextrin will reduce the pH of the cheese (Figure 2: pH of cheese analogue). This is similar to [26] which states that the addition of maltodextrin up to 15% will reduce the acidity in instant beverage products. Maltodextrin contains many hydroxyl (OH) groups that were able to neutralize the acidity properties of the products. The higher concentrations of the maltodextrin will be increasing the pH so that it would help to reduce the sour taste.

To get the maximum yield and total solids, the pH should be adjusted to reach the isoelectric point (4.6-4.7). However, in this study the pH was not adjusted to the isoelectric point since the adjustment in this study was based on the amount of citric acid added. If too much citric acid was added, it was feared that the cheese would be too sour.

**Figure 3:**
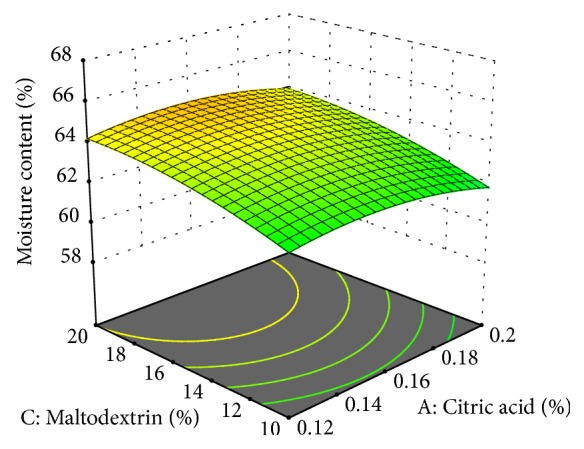
Moisture content of cheese analogue was affected by concentration of citric acid and maltodextrin.

The moisture content of cheese analogue was 58.725-66.103% (Figure 3: moisture content of cheese analogue). The moisture content of the cheese analogue in this study is in accordance with [9] which states that the cheese spread analogue has a moisture content of 55-80%.

The concentration of maltodextrin was directly proportional to the moisture content. It means that the higher the concentration of maltodextrin, the higher the moisture content. This was due to the ability of maltodextrin to bind water in a substance. This result is in accordance with [24] which states that the higher the concentration of maltodextrin added to food, the higher the moisture content.

The more addition of acid in cheese production would decrease the moisture content in the cheese. The addition of acid would accelerate the pH reduction. Low pH makes the condition more acidic, so the separation between whey and curd would become easier. This way of separating the whey and curd would result in lower moisture content in the curd. Therefore, the moisture content in the cheese was also lower [27].

Papain concentration did not affect the moisture content of the cheese spread analogue. This result is slightly different from [25] which states that the higher concentration of papain added, the lower moisture content. According to [25], in the cheese making from cow's milk, the higher amount of papain added will decrease the moisture content since papain ability to bind water will also decrease.

The dissolved protein of cheese analogue was 11.16-21.63%. The dissolved protein in this study is similar to [25], which is 12.14-22.56%. In their study, the cheese analogue is made from cow's milk which is processed by adding papain. Thus, although there was difference in the raw material used, the dissolved protein in cheese analogue made from corn milk and cow's milk processed using the same coagulant (papain) was the same.

The addition of papain will increase the dissolved protein in cheese analogue. Papain is a protease enzyme; thus the more papain added would increase the proteolytic activity. An increase in proteolytic activity will help the process of breaking down protein into peptides and amino acids, so that the level of dissolved protein in cheese analogue will increase [14].

The addition of citric acid would increase the content of dissolved protein. The addition of citric acid served to create an acidic condition so that isoelectric point of 4.6–4.7 was achieved. At the isoelectric point, the process of protein coagulation takes place so that* curd* can be separated from* whey* during the coagulation [28]. However, in the making of this cheese analogue, the pH of the cheese did not reach the isoelectric point so that the separation between whey and curd was not optimum since some of the protein was dissolved in the whey.

**Figure 4:**
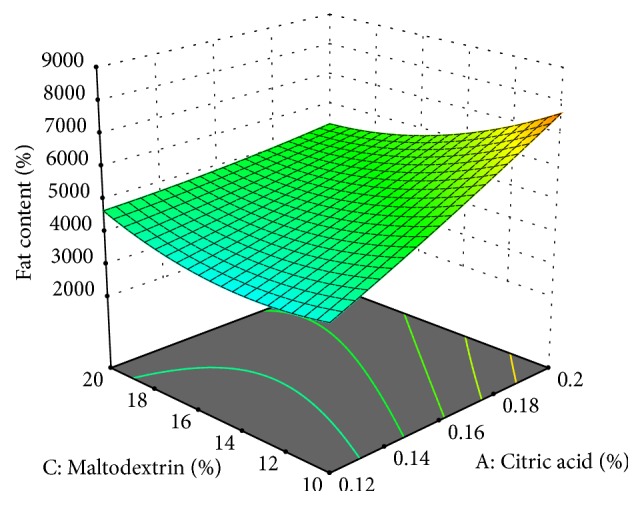
Fat content of cheese analogue was affected by concentration of citric acid and maltodextrin.

The fat content of the cheese analogue was 2.095-8.425% (Figure 4). The fat content of the cheese was lower than the cheese in general which is 20.5-24.2% [29]. This was because the raw material used was corn extract which has lower fat content than cow's milk. In addition, the process of cheese making in this study did not involve the ripening process. Thus, there was possibility that there might be fat dissolved in* whey* during pressing. The low fat content of the cheese was preferred by some people who wanted to consume low-fat cheese. Thus, the low fat content of the cheese became the advantage of this cheese analogue.

The more the citric acid added, the greater the fat content produced (Figure 4). This result is similar to [18] which states that increasing the concentration of acid added will also increase the fat content produced. This was because citric acid could increase the fat binding capacity in the cheese spread analogue. According to [19], the addition of citric acid can cause the denaturation of protein in which it will accelerate the coagulation process which causes the fat inability to come out because the protein is in the outer layer of fat globules. The rapid coagulation process by this acid has caused the fat binding capacity to increase along with the addition of citric acid.

The addition of papain in large amount would cause the fat content of cheese produced to decrease. This was due to the denaturation of protein complexes that caused the fat globule membrane to break and lead to fat leakage. In addition, larger fat globules would be easier to come out of the curd during the pressing. There are two possibilities that might occur in the process of cheese making: i.e., the fat would be lost in the whey or fat would fill the curd cavities. According to [30], the protein is on the outer layer of fat globule. The higher protein content contained in cheese meant that there would be more of fat that could be bound and maintained in cheese, so that the fat content would be higher.

The addition of maltodextrin did not significantly affect the fat content of cheese analogue. This was because maltodextrin has a hygroscopic property, so maltodextrin has the ability to bind water. On the other hand, fat has hydrophobic property and is insoluble in water. Thus, the tendency for maltodextrin to react with fat is very small [28]. The addition of maltodextrin in food was usually intended as filling material that could increase the yield and total solids of the food. Maltodextrin could also be used as emulsifier, to coat the flavor component and increase the volume.

### 3.2. Formula Optimization and Verification of the Optimum Formula

The purpose of using Response Surface Methodology for formula optimization was to find the best conditions that could bring together all the objective functions [31]. The components of citric acid, papain, and maltodextrin were optimized with the targets range of 3 since it served as the factor tried that would be used in a certain range. Moisture content is optimized with target range of 3 since the moisture content of the cheese analogue was 58.725-66.103%, which was still within the range of moisture content in fresh cheese (55-80%) [32]. The yield and protein content were optimized with the maximum target range of 5 since it was adjusted to the purpose of producing cheese analogue with the highest yield and protein content. pH was optimized with the target range of 3 so that the cheese analogue produced would be less sour, but still around the isoelectric point to obtain the maximum yield. The fat content of cheese analogue was 2.095-8.425% and was optimized with the minimum targets range of 3 since cheese analogue was expected to contain less fat.

From the optimization stage, there were 3 optimum formulas obtained. First, the optimum formula I was made with the addition of 0.2% citric acid, 0.029% papain, and 20% maltodextrin. Second, the optimum formula II was made with the addition 0.2% citric acid, 0.029% papain, and 19.995% maltodextrin. Third, the optimum formula III was made with the addition of 0.2% citric acid, 0.029% papain, and 19.912% maltodextrin. These three optimum formulas had the same concentration of citric acid and papain, but the concentration of maltodextrin was different.

Each formula has been proven to be in accordance with the predictions (Table 1). The moisture content of cheese analogue (61.43-62.08%) was higher than other cheese spread analogues, i.e., 41.1%-54.3% [25]. The other properties (yield, dissolved protein, and pH) were almost the same as cheese in general, while the fat content was lower.

### 3.3. The Characteristics of the Best Product

The three optimal formulas were analyzed by its sensory properties using rank tests. The sensory properties tested were color, aroma, taste, spread ability, texture, and level of preference. From the test result on the color, the best formula obtained was formula I, i.e., with the addition of concentration of citric acid 0.20%, papain 0.029%, and maltodextrin 20%. This was because the concentration of maltodextrin in formula I was higher compared to that in formulas II and III. According to [33] maltodextrin is a starch hydrolysis product which has a slightly yellowish-white color. The addition of maltodextrin in large amount would make the intensity of the yellow color of the cheese fade.

The criterion for the best cheese based on its aroma means that it had the most distinctive cheese aroma. Based on the test result, the best formula to create the distinctive aroma was formula I (with the addition of citric acid 0.20%, papain 0.029%, and maltodextrin 20%). One of the functions of maltodextrin was as coating ingredient, so the addition of maltodextrin to the production of this cheese analogue would coat the flavor component and reduce the loss of volatile compounds.

The criterion for the best cheese based on its flavor was that the flavor of cheese should not be too acidic or sour. According to the panelists' assessment, cheese analogue produced using formula I has the best flavor. This was because the concentration of maltodextrin added in formula I was higher compared to formulas II and III. Maltodextrin has a bland and slightly sweet taste and is hygroscopic, so it is able to reduce the acidic (sour) taste in the cheese produced [24].

The best spreadable criterion for cheese spread analogue was that the cheese can be spread easily on the food (bread was used in this study). From the test results, the best spread ability of the cheese was obtained from formula I, i.e., with the highest addition of maltodextrin (20%). This result is consistent with [34] which states that maltodextrin has a high solubility, is plastic, and is able to form films and form a body. The more maltodextrin is added to the cheese, the easier it is to be spread. According to [35], maltodextrin has the ability to form gels and bind water so it is often used to improve the texture quality (making the texture of the food softer), as an emulsifier, gelation, and water storage, and as a substitute for fat so that the product has a softer texture.

The test result on the level of preference showed that the best formula obtained was formula I. The assessment of preference level by the panelists was a combination of some properties that were considered to have the best value or rank.

## 4. Conclusions

The optimum formula to produce cheese spread analogue from sweet corn with the highest protein content and yield was with the addition of citric acid 0.20%, papain 0.029%, and maltodextrin 20%. The cheese analogue produced using the optimum formula had a moisture content of 61.590%, yield of 17.512%, dissolved protein of 19.837%, pH of 5.4, and fat of 6.976%. The sensory characteristics of cheese analogue spread from sweet corn extract are similar to cheese from cow's milk, i.e., a yellowish-white color, distinctive aroma of cheese, no taste sour, soft texture, and ease of spreading. Therefore, it was possible to explore the sweet corn as ingredient of spread cheese, especially for its low fat content.

## Figures and Tables

**Table 1:** The verification results of the optimum formula.

	Formula I			Formula II			Formula III		
Response	Actual Data	Predictive value	Verification	Actual Data	Predictive value	Verification	Actual Data	Predictive value	Verification
Moisture Content (%)	61.590	64.004	Appropriate	61.426	64.003	Appropriate	62.083	64.001	Appropriate
Yield (%)	17.512	17.484	Appropriate	16.144	17.376	Appropriate	15.888	17.367	Appropriate
Dissolved solids (°Brix)	19.00	19.112	Appropriate	9.00	17.367	Appropriate	18	19.065	Appropriate
Dissolved protein (% db)	19.837	19.784	Appropriate	19.269	19.859	Appropriate	18.806	19.856	Appropriate
pH	5.4	5.365	Appropriate	5.5	5.369	Appropriate	5.5	5.370	Appropriate
Fat (% db)	6.976	5.420	Appropriate	7.731	5.399	Appropriate	7.288	0.747	Appropriate

## Data Availability

The data used to support the findings of this study are available from the corresponding author upon request.
